# Magnesiothermic synthesis of sulfur-doped graphene as an efficient metal-free electrocatalyst for oxygen reduction

**DOI:** 10.1038/srep09304

**Published:** 2015-03-20

**Authors:** Jiacheng Wang, Ruguang Ma, Zhenzhen Zhou, Guanghui Liu, Qian Liu

**Affiliations:** 1 State Key Laboratory of High Performance Ceramics and Superfine Microstructure, Shanghai Institute of Ceramics, Chinese Academy of Sciences, Shanghai 200050, P. R. China; 2 Innovation Center for Inorganic Materials Genomic Science, Shanghai Institute of Ceramics, Chinese Academy of Sciences, Shanghai 200050, P. R. China; 3 Shanghai Institute of Materials Genome, Shanghai, P. R. China

## Abstract

Efficient metal-free electrocatalysts for oxygen reduction reaction (ORR) are highly expected in future low-cost energy systems. We have successfully prepared crumpled, sheet-like, sulfur-doped graphene by magnesiothermic reduction of easily available, low-cost, nontoxic CO_2_ (in the form of Na_2_CO_3_) and Na_2_SO_4_ as the carbon and sulfur sources, respectively. At high temperature, Mg can reduce not only carbon in the oxidation state of +4 in CO_3_^2−^ to form graphene, but also sulfur in SO_4_^2−^ from its highest (+6) to lowest valence which was hybridized into the carbon sp^2^ framework. Various characterization results show that sulfur-doped graphene with only few layers has an appropriate sulfur content, hierarchically robust porous structure, large surface area/pore volume, and highly graphitized textures. The S-doped graphene samples exhibit not only a high activity for ORR with a four-electron pathway, but also superior durability and tolerance to MeOH crossover to 40% Pt/C. This is mainly ascribed to the combination of sulfur-related active sites and hierarchical porous textures, facilitating fast diffusion of oxygen molecules and electrolyte to catalytic sites and release of products from the sites.

Because of the predicted detrimental effects (*e.g.*, global climate warming) of CO_2_ emission, there is growing interest in designing novel materials and technologies for carbon capture and storage, as well as chemical fixation of CO_2_ in constructing a future low-carbon global economy. CO_2_ emitted from fuel-fired power plant is captured by a well-developed chemical absorption process using aqueous amine-ammonia solutions in industry^1^ and physisorption between solid adsorbents (*e.g.* zeolites^2^, mesoporous silicas^3^, activated carbons^4^,^5^,^6^, metal-organic frameworks (MOFs)^7^, *etc*) and CO_2_ molecules^8^ in laboratory research.

Except for CO_2_ capture and storage, chemical utilization of CO_2_ is another major concern with respect to the reduction of CO_2_ emission into the atmosphere. At the same time, it can exploit CO_2_ as a non-toxic, economical, environmentally friendly, and abundant carbon feedstock for the synthesis of useful chemical compounds. It has been reported that CO_2_ could be used as raw materials to produce various valuable compounds including organic carbonates^9^, urea derivatives^10^, formic acid^11^, methanol^11^,^12^, carbon monoxide^13^, and even diamond^14^, *etc*^15^, in the presence of catalyst/reaction media. Especially, CO_2_ could also be reduced into the few-layer graphene nanostructure with different morphologies^16^,^17^. The dye sensitized solar cell with CO_2_-derived graphene as a counter electrode shows a high energy conversion efficiency of 8.1%, 10 times higher than that (0.7%) of a cell with the conventional graphene *via* chemical exfoliation of graphite^17^. Pure graphene with 3D honeycomb-like microstructure was prepared by the reaction of carbon monoxide and lithium oxide, reported by Hu and his co-workers^18^. Herein, we describe a one-pot magnesiothermic reduction strategy to transform CO_2_ (in the form of Na_2_CO_3_) in the presence of Na_2_SO_4_ as an inorganic sulfur source to graphitized, wrinkled, sulfur-doped graphene with only few layers.

Since the seminal work of Geim and co-workers on the preparation of single layer graphene (sp^2^-hybridized carbon)^19^, immense excitement has ensued, with graphene and modified graphene structures being shown to have notable and potentially exploitable electronic^18^,^19^, optical^20^, catalytic^21^,^22^, physical properties^23^,^24^, and others^25^,^26^. Both theoretical and experimental studies have shown that the doping of heteroatoms (*e.g.* nitrogen (N, electronegativity: 3.04), phosphorus (P, electronegativity: 2.19), sulfur (S, electronegativity: 2.58), boron (B, electronegativity: 2.04), *etc*) with distinct electronegativity with respect to carbon atoms (electronegativity: 2.55) into sp^2^-hybridized carbon frameworks can remarkably modify their electronic structures and chemical activities as well as the Fermi level of the host. This strategy is simple and efficient to improve the properties of graphene. At the same time, the robust framework and most of the intrinsic properties of graphene are maintained after the doping of the heteroatoms. Chemical vapor deposition (CVD) and the solvothermal pathway have been adopted to produce doped graphene using liquid precursors^27^,^28^. Furthermore, the synthesis of heteroatom-doped graphene from natural graphite generally needs two steps: (I) the oxidation of graphite resulting in the formation of graphene oxide (GO); (II) post-reacting GO with various N-, P-, or B-containing precursors (*e.g.* urea^29^, ammonia^30^, tribenzylphosphine^31^, boric acid^32^, *etc*). Compared to N- (or B-) doped graphenes, little attention has been paid to the doping of S atoms into the graphene framework^33^,^34^,^35^, although S-doped graphene has great potential as the electrocatalyst for oxygen reduction reaction (ORR), which is of high industrial importance in fuel cells.

S-containing functionalities have been successfully incorporated into the sp^2^ carbon lattice by two procedures: (I) CVD technique using liquid organics and (II) the pyrolysis of GO and S-containing precursors. For example, Gao and co-workers reported the formation of S-doped graphene on Cu foil using liquid S-hexane mixture vapor at 950°C^36^. However, the S content is as low as 0.6 at% and most of sulfur atoms exist in the linear nanodomain (2 ~ 4 nm) other than doped S atoms. The pyrolysis of BDS and GO at 600 ~ 1050°C leads to S-doped graphenes with 1.3–2.1 at% S atoms^33^,^37^. The resulting S-doped graphene shows good electrocatalytic activity for ORR^38^ and it is also used as the support of synthesizing ultrathin Pt nanowires demonstrating higher catalytic activities toward methanol oxidation reaction compared with commercial Pt/C^37^. Thermal exfoliation of GO in H_2_S, SO_2_ or CS_2_ gas is an efficient procedure of doping graphene lattice with S atoms^34^,^39^, and the extent of S doping is evidently dependent on the type of GO used than the type of S gas atmosphere utilized during the synthesis^35^. The H_2_S-treated GO at 500 ~ 900°C has the content of 1.2–1.7 at% S, which was employed as metal-free electrocatalysts for ORR^34^. Furthermore, S- and N-dual-doped graphene materials were also prepared from GO using BDS/thiourea/thiophene as the S source and melamine/urea/pyrimidine as the N source, respectively^40^,^41^,^42^, or ammonium thiocyanate as the N/S dual-containing precursors^43^. Also, the synthesis of S-doped graphene using expensive GO (Sigma-Aldrich, product number: 763713, Price: 234 GBP/g) and toxic S-containing organics/gas in the reduced of forms rather than the oxidized forms generally abundantly present in nature is practically exclusively done at present. At the same time, the use of harmful S-containing (H_2_S, SO_2_ and CS_2_) gas flow as the S source could result in the production of large amount of exhaust waste. It is, however, yet a big challenge to directly generate S-doped graphene from easily available, low-cost, nontoxic Na_2_CO_3_ and Na_2_SO_4_ inorganic oxide salts as the C and S sources, respectively.

Herein, we have employed a one-step magnesiothermic reduction strategy to transform greenhouse gas CO_2_ (in the form of Na_2_CO_3_) in the presence of small amount of Na_2_SO_4_ as an inorganic S source to form S-doped graphene, as shown in Figure 1. At high temperatures, Mg metal could reduce carbon in the oxidation state of +4 in CO_3_^2−^ to form black graphene products. At the same time, the SO_4_^2−^ ions could supply S atoms to build C-S sp^2^ bonds integrated into the graphene lattice; S in its highest oxidation state of +6 in SO_4_^2−^ is reduced to the lowest valence by Mg metal. It is observed that S-doping has a great influence on the morphologies of graphene sheets. Thus, the present novel procedure can efficiently reduce the CO_2_ concentration in the atmosphere by chemically fixing carbon in CO_2_ to valuable doped, high-quality graphene. We also showed that the as-formed S-doped graphene materials demonstrate high electrocatalytic activity for ORR with a dominant four-electron reaction pathway, as well as excellent durability and MeOH crossover compared to commercial Pt/C catalyst. Thus they can be considered as ideal candidates as metal-free ORR electrocatalysts.

## Results

**Figure 2:**
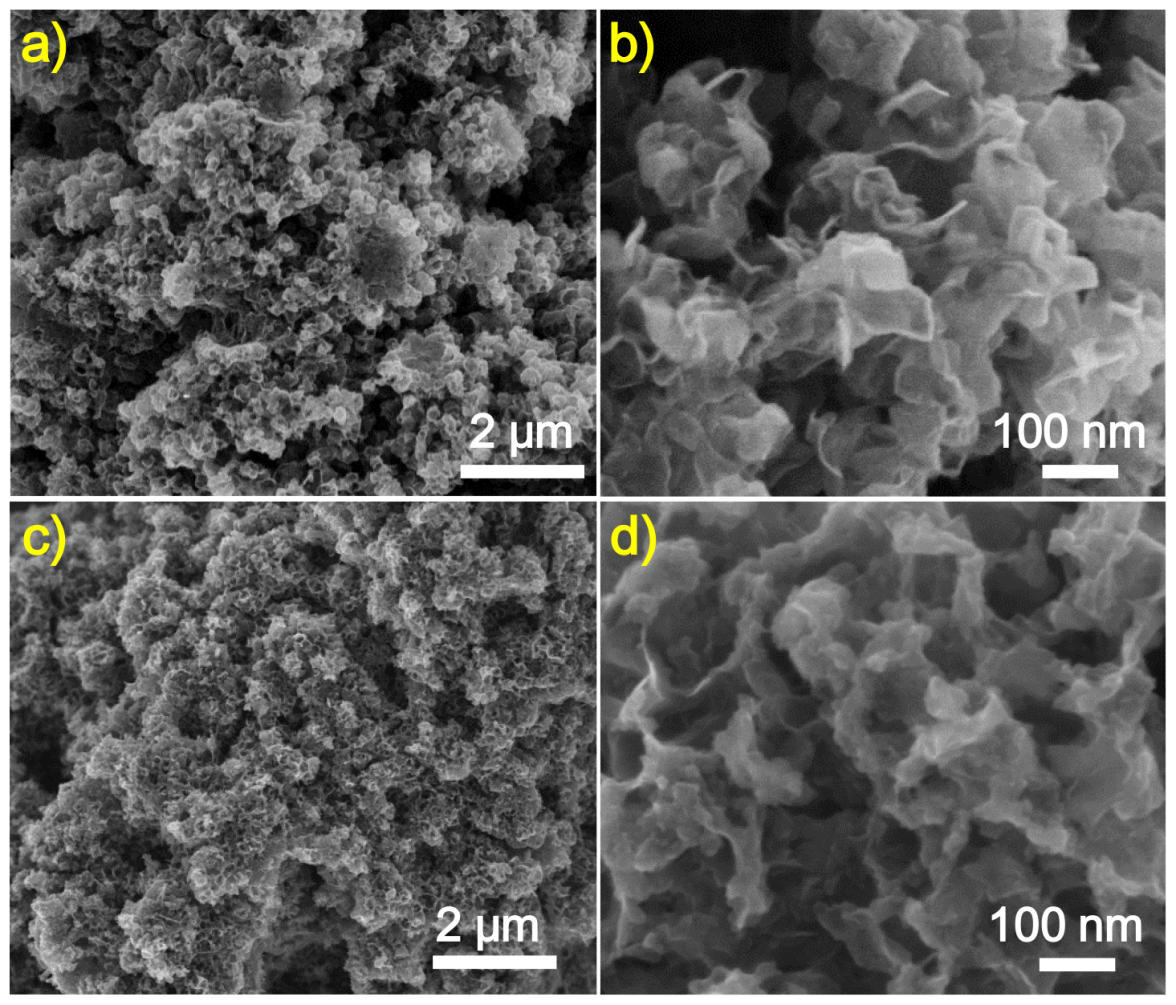
SEM images of CG-800 (a, b) and SG-800 (c, d).

After the pyrolysis of the mixture of Mg powder and Na_2_CO_3_, the product has a dark black color, suggesting the formation of a graphitic structure. Figure 2a–b show the morphologies of pure carbon-based graphene (CG) prepared by pyrolysing the physical mixture of Mg and Na_2_CO_3_ at 800°C (CG-800), observed by scanning electron microscopy (SEM). Low-magnification SEM image shows that CG-800 has a loose, porous structure (Figure 2a). At high magnification, CG-800 is constituted of many crumpled and wrinkled sheets with sharp edges (Figure 2b). The morphologies of these sheets with sizes of 100 ~ 200 nm are similar to those of sheet-like petals forming a flower. The yield of CG-800 is 55% calculated by the ratio of moles of graphene to Na_2_CO_3_. This yield decreases a little to 48% when using Na_2_SO_4_ as a sulfur source added into the physical mixture to produce S-doped graphene (SG-800). SG-800 exhibits a similar morphology to that of CG-800, confirmed by low-magnification SEM image (Figure 2c). However, high-resolution SEM image indicates that SG-800 is porous and composed of irregular sheets and particles without any sharp edges (Figure 2d). This finding suggests that sulfate added took part in the reaction, and thus changed the morphologies of the resulting S-doped graphene nanostructures. The sheet-like graphene nanostructures were also observed in those prepared by the carbonization of glucose in molten salts^38^,^44^.

**Figure 3:**
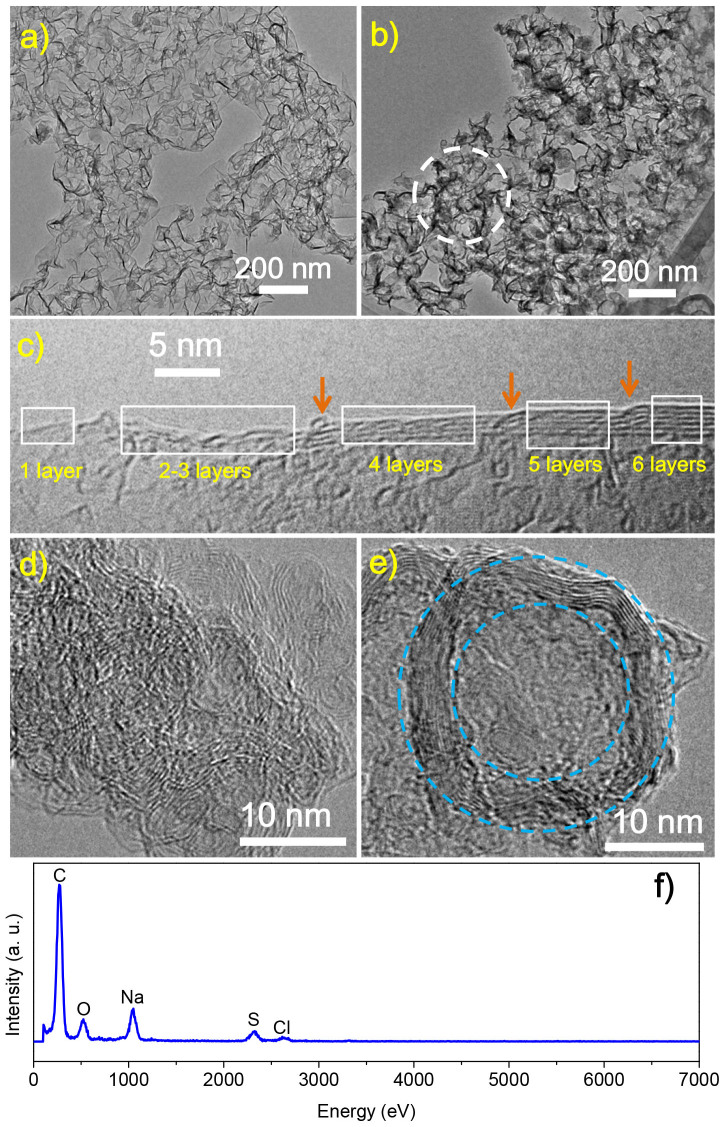
TEM images of CG-800 (a) and SG-800 (b), high-resolution TEM images of CG-800 (c) and SG-800 (d, e), and EDS analysis (f) on the circle area marked in the image (Figure 3b) of SG-800. The arrows in the image (c) indicate the formation of the steps due to the increment of graphene layers at the edge of graphene nanosheet.

Further observation on the morphology difference between pure graphene and S-doped graphene was confirmed by transmission electron microscopy (TEM). As shown in Figure 3a (also Supplementary Figure S1), it is clear that CG-800 is composed of large, perfect two-dimensional (2D) sheets with the crumples, implying a 2D sp^2^-hybridized nature. At the edge of the sheet, high-resolution TEM images clearly exhibit the signature image of the few-layer graphene with the number of lower than 10 layers (Figure 3c and Supplementary Figure S1). In Figure 3c, the steps are formed at the edge of a graphene sheet due to the increment of graphene layers (from 1 to 6 layers). The morphologies of CG-800 prepared using Na_2_CO_3_ as the carbon source are different from those of graphenes prepared using CO_2_ or CaCO_3_ as the carbon source^16^,^17^,^45^, implying the great influences of different carbon sources on final graphene materials. Moreover, the introduction of sulfate salt also significantly changed the morphologies of graphene products, as shown in Figure 3b (also Supplementary Figure S2). The resulting SG-800 demonstrates crumpled, porous structures which are distinct from CG-800. In most areas, the hollow spherical graphene nanostructures with more distorted morphology can be clearly observed in SG-800 (Supplementary Figure S2). It is possible that Na_2_SO_4_ acts as not only the S source but also the ‘salt template' promoting the synthesis of porous graphene materials by adjusting the growth direction of graphene layers on the surface of salt particles^46^. High-resolution TEM images exhibit the fringe-like graphene rings with few layers in SG-800 (Figure 3d–e). The measured interlayer distances are in the range of 0.34–0.38 nm, a little larger than the *d*-spacing of (002) crystal plane (0.335 nm) of bulk graphite due to slight expansion and distortion^47^,^48^. Successful doping of S atoms into the graphene layers prepared by magnesiothermic reduction was confirmed by energy-dispersive spectrometry (EDS). The EDS spectrum of SG-800 indicates the presence of S (2.2 *at*%) as well as O (3.6 *at*%) element in the graphene layers. Also, small amount of Na and Cl elements were found, which are mainly due to the trapped Na compounds between the graphene layers during the magnesiothermic reaction and the following acid-washing step^16^.

**Figure 4:**
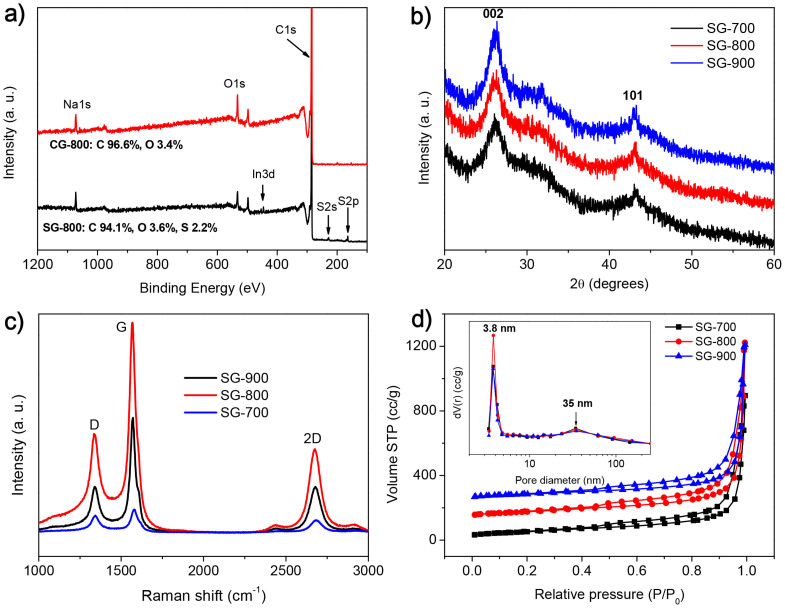
XPS survey spectra (a) of CG-800 and SG-800, and wide-angle XRD patterns (b), Raman spectra (c), and nitrogen sorption isotherms (d, inset: pore size distributions) of S-doped graphenes. Indium (In) 3d peaks in Figure 4a is ascribed to the In substrate supporting graphene samples for XPS measurements.

In accordance with EDS analysis, X-ray photoelectron spectroscopy (XPS) survey scans of S-doped graphenes prepared at different temperatures imply the varied S contents of 1.8 ~ 2.6 *at*% (Figure 4a and Supplementary Table S1). The S content in SG-800 obtained by XPS analysis is close to that obtained from the EDS result, suggesting that S functional groups are homogenously distributed throughout the sample. And an evident signal of Na 1s is ascribed to the trapped Na elements within the graphene layers (Figure 4a)^16^. After the ultrasonication of SG-800 in the solvent, XPS analysis showed that the S signals have no evident changes, implying the formation of the covalent C-S bonds in these S-doped graphenes. The S contents in present graphene samples are evidently higher than those of S-doped graphenes (S: *ca.* 0.5 ~ 0.6 at%) prepared by the pyrolysis of GO and BDS^33^ and a CVD technique using liquid organic^49^, and comparable to that of N, S-dual-doped graphene (S: 1.3 ~ 2.0 at%) prepared by annealing GO with BDS or H_2_S at high temperatures^34^,^42^. As shown in Figure 4b and Supplementary Figure S3, all wide-angle X-ray diffraction (XRD) patterns of both pure graphenes and S-doped graphenes prepared at 700–900°C are very similar to that of typical sp^2^-bonded carbons, exhibiting that all of them possess a crystalline graphene structure. Indeed, two strong and broad characteristic peaks can be observed at 26.1 and 43.3 degrees arising from the (002) and (100) planes of graphene sheets, respectively^50^. The calculated *d*-spacing of (002) crystal plane is about 0.35 nm, slightly larger than the interlayer spacing in graphite (0.335 nm). These results match well with those from TEM analysis, implying the formation of high-quality graphene structures in SG-800. In contrast, magnesiothermic reduction of CaCO_3_ at 800°C led to the production of amorphous carbons rather than crystalline graphene^45^, implying Na_2_CO_3_ is better carbon source for the synthesis of graphene nanostructures.

Further structural information about S-doped graphenes was obtained from Raman spectroscopy technique through the determination of the G (related to pristine sp^2^ graphitic layer) bands at ~1560 cm^−1^, D (related to defect in sp^2^ lattice) bands at ~1350 cm^−1^, and 2D bands at ~2725 cm^−1^ in the spectrum (Figure 4c and Supplementary Figure S4). The intensity of the D to G band (*I*_D_/*I*_G_) is used to evaluate the defect content of the graphene. As shown in Supplementary Table S2, the reaction temperature higher, the values of *I*_D_/*I*_G_ for the resulting S-doped graphenes lower. For example, the value of *I*_D_/*I*_G_ is 0.75 for the sample prepared at 700°C, and it decreases to 0.47 for SG-800, and further to 0.41 for SG-900. This implies that a higher reaction temperature is favorable for defect repair to form graphene with more perfect layers. Furthermore, it can be found that the *I*_D_/*I*_G_ values of the resulting graphene materials are evidently higher than those of most chemically reduced GO^51^, which implies that the present magnesiothermic reduction route is better than the chemical methods to prepare high-quality graphenes with less defects. Moreover, the S doping of the graphene layers also can be proved by Raman data. Under the similar synthesis conditions, the G peak (1574 cm^−1^) of CG-800 is shifted down to 1570 cm^−1^ for SG-800, which is an important characteristics of *n*-type doping of graphene by the substitution. The similar red shift is also reported for N-doped graphene^49^,^52^. The 2D band is an overtone of the D band, and its position is highly correlated with the layer numbers of the graphene sheets^53^. The 2D peaks (2676–2685 cm^−1^) for S-doped graphenes are significantly lower than 2725 cm^−1^ for pure graphite, showing the successful preparation of few-layer graphene nanostructures in all samples. When increasing reaction temperatures, the 2D position (2685 cm^−1^) for SG-700 shifts down to 2678 cm^−1^ for SG-800, and 2676 cm^−1^ for SG-900, respectively. It indicates that SG-800 and SG-900 have similar average numbers of the graphene nanosheets, both of which are smaller than the average numbers for SG-700.

To get insights in pore structures of the resulting graphenes prepared by magnesiothermic reduction, the N_2_ adsorption-desorption isotherms at −196°C were measured, which allow the calculation of Brunauer–Emmett–Teller (BET) surface area, total pore volume, and pore size distribution. The corresponding nitrogen adsorption-desorption isotherms and pore size distributions for S-doped graphenes and pure graphenes are shown in Figure 4d and Supplementary Figure S5, respectively, and the textural properties are listed in Table 1. All isotherms exhibit a similar type IV curve with a H3 hysteresis loop, implying that the reaction temperatures have no evident impact on the porosity and pore microstructure of the resulting graphene materials. The calculated specific surface areas and pore volumes of three pure graphenes are 222–239 m^2^/g and 1.887–1.936 cm^3^/g, respectively. These data slightly decrease to 188–199 m^2^/g and 1.383–1.604 cm^3^/g, respectively, for S-doped samples compared with those of pure graphenes. Among these S-doped graphenes, the sample prepared at 800°C demonstrates the largest specific surface area and pore volume. The specific surface areas of these graphenes are significantly lower than the theoretical surface area of 2630 m^2^/g for individual isolated graphene sheets^54^, but they are comparable to those of N-doped thermally reduced GO^55^. The graphenes hardly adsorb nitrogen in the low relative pressure range, implying no micropores in these samples. This fact also hints that the resulting graphene layers are perfectly crystallized and no micropores are formed on the graphene layers during the magnesiothermic reduction. The hysteresis loop is located in the relative pressure (P/P_0_) of 0.45–1.0, which might be a result of small mesopores and intersheet textural large pores. The pore size distribution curves of pure graphenes and S-doped graphenes calculated by the Barrett-Joyner-Halenda (BJH) model, show a single well-defined peak at 3.8 nm in all curves, implying that all graphene samples have narrow mesopores. It is noted that a weak peak is found at about 35 nm, possibly ascribed to the intersheet textural pores.

**Figure 5:**
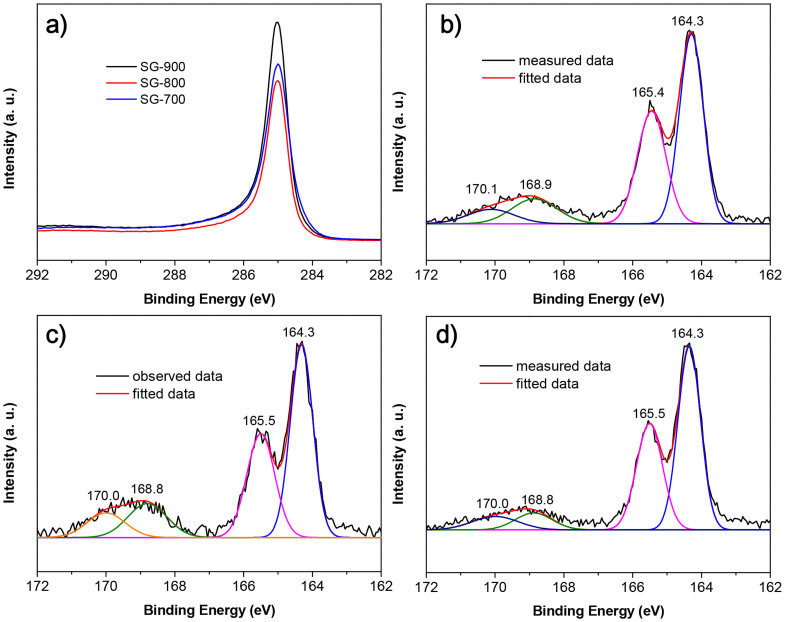
C 1s (a) and S 2p (b, SG-700; c, SG-800 and d, SG-900) XPS spectra of S-doped graphenes.

To further investigate the chemical environments of S and C atoms in the graphene layers, high-resolution X-ray photoelectron spectroscopy (XPS) spectra in the C1s and S 2p regions were recorded in Figure 5. The C 1s peak for S-doped graphene materials was observed at ~285.0 eV, which is consistent with graphene sp^2^ carbon in the samples. Furthermore, there are very minor shoulder contributions to the XPS signals at binding energies of 286.0–288.0 eV, which are assigned to sulfur- and/or oxygen-bound carbon atoms on the material surface^56^. The broad signals at 291.0–292.0 eV correspond to the π-π* shake-up peak^57^. The XPS spectra for S 2p in S-doped graphenes usually comprise of two parts. There are minor contributions (168.8–170.1 eV) of oxidized S species, such as sulfate and sulfonate functional groups. Another region (164.3–165.5 eV) is corresponding to spin-orbit splitting of S atoms doped into the graphene layers, *e.g.* S dominated in the graphene framework *via* the formation of the sulfide bridges (-C-S-C)^58^. Thus, it can be concluded that most of S atoms are directly doped into the carbon backbone of the materials. In terms of chemical bonding, S-doped graphenes are evidently different from sulfuric acid treated biomass-derived carbons in which all S atoms still have the oxidation of +6^59^. These results suggest that S in its highest oxidation state in Na_2_SO_4_ could be reduced to its lowest valence, which is then hybridized into the graphene layers during the magensiothermic reduction.

It has been reported that S-doped graphenes could act as metal-free electrocatalysts for ORR. In present work, it has been proved that the *in-situ* reactive doping of S into the graphene framework could be accomplished by magnesiothermic reduction of Na_2_CO_3_ in the presence of Na_2_SO_4_ as an inorganic S source. It is believed that Mg could reduce not only CO_3_^2−^ to the graphitized carbons, but also S in the highest oxidation state of +6 in SO_4_^2−^ to the lowest valence which was further hybridized into the carbon sp^2^ framework. The addition of Na_2_SO_4_ during the magnesiothermic reduction evidently not only affects their structure and morphologies, but also improves the electrocatalytic performance of the resulting S-doped graphenes for ORR as discussed below. The present S-doped graphenes possess an appropriate S content, hierarchical porous textures, and highly graphitized structure. Such unique features will promote fast access of oxygen molecules to the catalytic sites and can favor the swift diffusion of electrons in the electrode during the ORR process.

**Figure 6:**
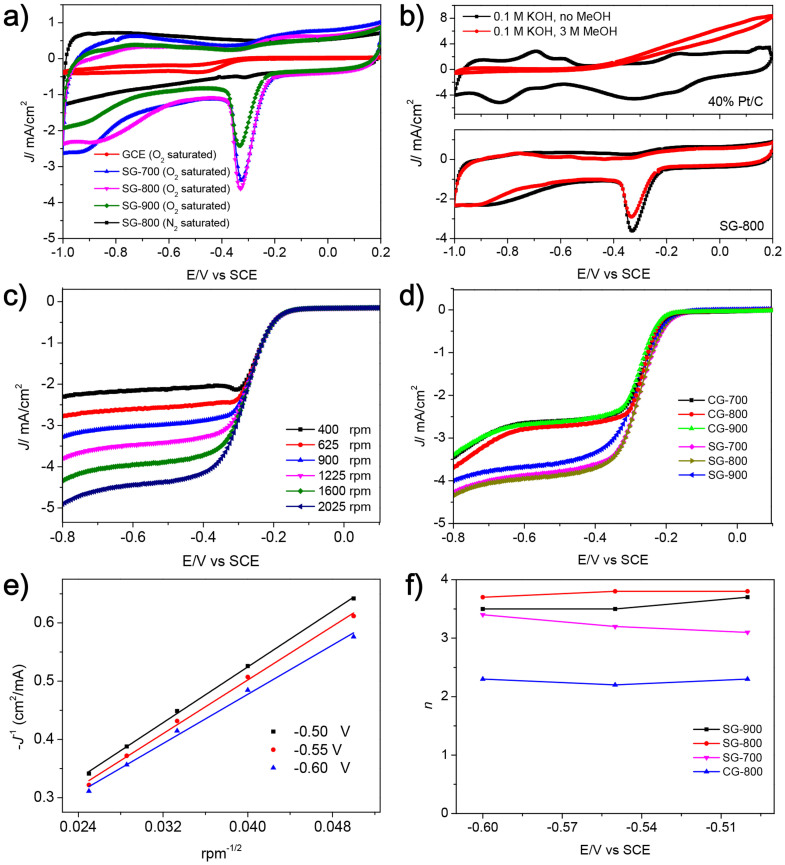
Electrochemical ORR catalytic performance of samples. (a) CV curves of blank glassy carbon electrode (GCE) and S-doped graphenes in O_2_-statured or N_2_-saturated 0.1 M KOH aqueous solution (sweep rate: 50 mV/s). (b) Comparison of CV curves of SG-800 and 40% Pt/C in O_2_-saturated 0.1 M KOH solution upon the addition of MeOH (3 M). (c) Linear-sweep voltammograms (LSVs) of SG-800 in O_2_-saturated 0.1 M KOH solution at different rotating speeds of 400 ~ 1600 rpm (sweep rate: 5 mV/s). (d) LSVs of different samples at 1600 rpm. (e) Koutecky-Levich (K-L) plots of SG-800 at different potentials (*vs*. SCE). The lines are drawn by linearly fitting the corresponding dots. (f) The electron transfer numbers (*n*) at SG-800 and CG-800 calculated based on the K-L equations in the potential range of −0.5 ~ −0.6 V (*vs*. SCE).

To assess the electrocatalytic properties of the resulting S-doped graphenes, the ORR catalytic activities were evaluated using a three-electrode electrochemical station. Figure 6a shows the cyclic voltammogram (CV) curves of a bare glassy carbon electrode (GCE) and S-doped graphenes deposited on a GCE in N_2_- and O_2_-saturated 0.1 M KOH aqueous electrolyte at a scan rate of 50 mV/s. It was found that ORR on a GCE requires large over potentials (peak potential of −0.49 V *vs*. saturated calomel electrode (SCE)) with very low oxygen reduction current density (−0.38 mA/cm^2^); this reduction current was reported to be the two-electron reduction process of O_2_ to peroxide which is electrochemically mediated by the oxygen-containing groups at the surface of bare GCE. The CVs for S-doped graphenes exhibit nearly rectangular shapes, showing high conductivity with superior capacitive current. A well-defined oxygen reduction peak at ~−0.32 V (*vs*. SCE) is observed for S-doped graphenes in O_2_-saturated 0.1 M KOH solution, while there is no such a peak in N_2_-saturated one, indicating that O_2_ is reduced on S-doped graphenes (Figure 6a). SG-800 demonstrates the highest current density of 3.68 mA/cm^2^ among three samples, indicating a pronounced electrocatalytic activity of SG-800 for ORR.

To examine the possible crossover effects, the electrocatalytic selectivity of SG-800 and 40% Pt/C against electro-oxidation of methanol (MeOH, a typical fuel molecule) by CVs in an O_2_-saturated 0.1 M KOH solution with 3 M MeOH. The cathode signals for ORR completely disappear in the CV curves for commercial 40% Pt/C electrode when the O_2_-saturated 0.1 M KOH solution was replaced by 0.1 M KOH solution with 3 M MeOH (Figure 6b). The current density over the potential range larger than 0 V (*vs*. SCE) significantly increases, corresponding to methanol electro-oxidation. In contrast, no noticeable change except for a slight decrease in the current density of ORR is found for SG-800 under the same conditions. These voltammograms show that S-doped graphenes have higher selectivity for ORR with a remarkable improved ability to avoid the cross effects than commercial Pt/C. It is mainly ascribed to the highly graphitized nanostructures, which increase both the electronic conductivity and corrosion resistance of SG-800. Thus, this kind of S-doped graphenes has great potentials as the electrocatalytic material in direct methanol and alkaline fuel cells.

The increased electrocatalytic activity of S-doped graphenes was further confirmed by linear sweep voltammetry (LSV) in an O_2_-saturated 0.1 M KOH aqueous solution at different rotating speeds on a rotating disk electrode (RDE) (sweep rate: 5 mV/s). The voltammetric profiles show that the current density is increased with an increase in rotating speeds (Figure 6c). The corresponding onset potential of SG-800 for the ORR is about −0.15 V, close to that identified from CV measurement (−0.17 V). In a N_2_-saturated 0.1 M KOH solution, the resulting ORR current density for SG-800 is neglectable (Supplementary Figure S6), indicating that O_2_ is reduced on S-doped graphene electrocatalyst. The LSVs of pure graphene, S-doped graphene, and 40% Pt/C in an O_2_-saturated 0.1 M KOH solution were measured at 1600 rpm (sweep rate: 5 mV/s). The S doping into the graphene framework evidently results in the positively shift of the onset potential from −0.18 V (*vs*. SCE) for pure graphene to −0.15 V (*vs*. SCE) for S-doped graphene. It implies the important role of S atoms doped into the graphene layers in adjusting chemical/electronic properties of graphene, thus improving the ORR catalytic performance of S-doped graphene. All S-doped graphene samples show the onset potentials are just negatively shifted by 0.12 V (*vs*. SCE) related to that (−0.04 V *vs*. SCE) for commercial Pt/C (Supplementary Figure S7). This value is evidently compared to other recently reported metal-free carbon-based electrocatalysts (*e. g.* N-doped graphene^29^,^60^, N-doped mesoporous carbon^61^, P-doped ordered mesoporous carbon^62^, N, S-dual-doped graphene^63^, *etc*) for ORR (Supplementary Table S3). Notably, the ORR current densities for all S-doped graphenes at −0.8 V (*vs*. SCE) are significantly higher than those for all pure graphenes, strongly indicating that S atoms have been doped into the layers of graphene sheets, and have similar impact to nitrogen atoms doped into the graphene layers on the electronic structure of doped graphenes. Among these graphene samples, SG-800 demonstrates the largest ORR current density (3.3 mA/cm^2^) at −0.32 V (the peak potential *vs*. SCE), which are evidently superior or compared to heteroatom-doped porous carbon-based electrocatalysts for ORR (Supplementary Table S3). The high ORR activity of SG-800 is ascribed to the appropriate amount of S atoms, highly graphitized carbon structures, and hierarchical porous textures. This unique structure also provides large number of proper channels for easy mass diffusion of electrolyte ions, oxygen molecules, and products.

By using the Koutecky-Levich (K-L) equation, we further analyzed the RDE data collected by LSVs. As shown in Figure 6e, the K-L plots of SG-800 in the potential range of −0.5 ~ −0.6 V (*vs*. SCE) exhibit good linearity, the slopes of which are approximately constant. It also indicates the first-order reaction kinetics with respect to the concentration of O_2_ dissolved. The exact electron transfer numbers of S-doped graphene and CG-800 were calculated according to the slopes of the linear fitted K-L plots on the base of the K-L equations as following^64^,
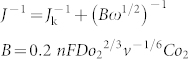
where *J* and *J*_k_ are the measured current density and kinetic-limiting current density, respectively, *n* is the electron transfer number, *F* is the Faraday constant (96485 F/g), *v* is the viscosity of the electrolyte (0.01 cm^2^/S), *C*_O2_ is the concentration of O_2_ (1.2 * 10^−6^ mol/cm^3^), and *D*_O2_ is the diffusion coefficient (1.9 * 10^−5^ cm^2^/s). The coefficient 0.2 is adopted when the rotating speed is expressed in rpm. As displayed in Figure 6f, the number of electrons transferred per O_2_ molecules (*n* = 3.1 ~ 3.8) in ORR is higher than two in the potential range of −0.5 V to −0.6 V, and SG-800 has the highest electron transfer numbers (3.7–3.8), possibly because of its highest surface area and pore volume and appropriate S content. This result indicates that the desirable four-electron reduction process for SG-800 to form water as the main product other than peroxide is predominant. In contrast, CG-800 has *n* value of *ca*. 2.2, which suggests that the two-electron reduction process with peroxide as the main product is dominant. Note that four-electron process is very important for ORR in fuel cells, because peroxides can poison the cell. This finding matches well with previous reports indicating that pure reduced GO can cause a two-electron pathway^40^. These results highlight the key role of doped sulfur atoms acting as active sites for promoting the four-electron process in ORR^33^.

**Figure 7:**
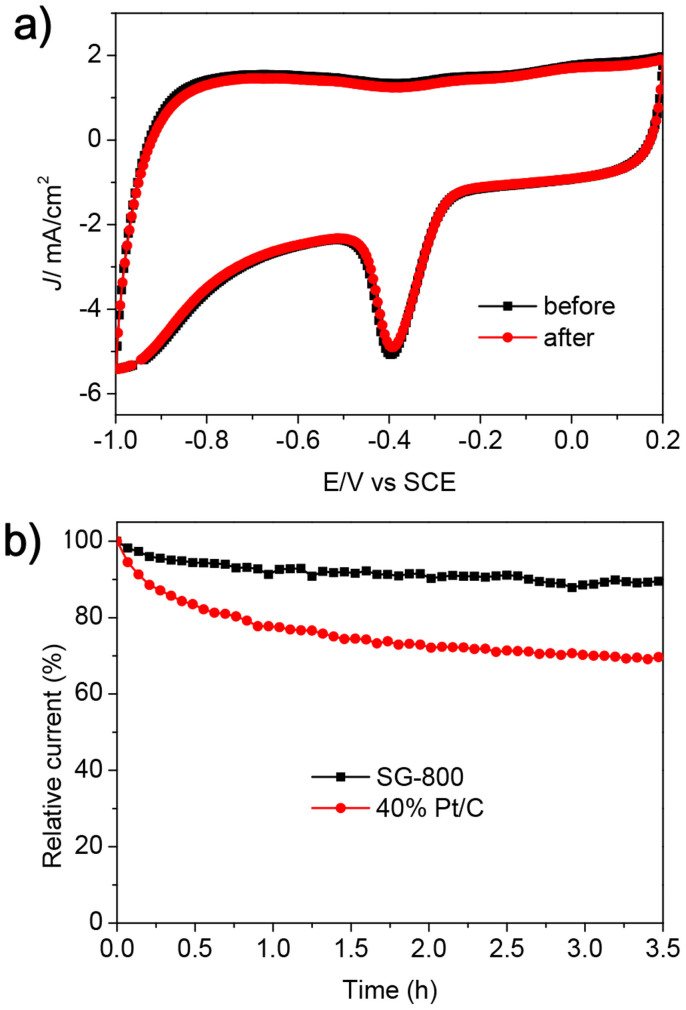
(a) CV curves of SG-800 electrode in O_2_-saturated 0.1 M KOH solution before and after a continuous potentiodynamic sweep for 4000 cycles with a scan rate of 200 mV/s. (b) Current-time (*i*-*t*) chronoamperometric response of SG-800 and commercial 40% Pt/C electrodes at -0.4 V (*vs*. SCE) in O_2_-saturated 0.1 M KOH solution at a rotating speed of 1600 rpm.

Another major concern is the stability of the S-doped graphene used as high-performance electrocatalyst for ORR. Accordingly, the continuous potential cycling to investigate the stability of SG-800 was performed. As shown in Figure 7a, Both the CV shape and current density have no any evident change after 4000 continuous cycles in the potential range of 0.2 ~ −1 V (*vs*. SCE) in O_2_-saturated 0.1 M KOH solution at a scan rate of 200 mV/s, implying the excellent capacitance stability of SG-800. Moreover, we also conducted a 3.5 h stability test for SG-800 and 40% Pt/C toward ORR using the *i*-*t* chronoamperometric response at a constant voltage of −0.4 V (*vs*. SCE) in a O_2_-saturated 0.1 M KOH solution at a rotating speed of 1600 rpm. As shown in Figure 7b, the current densities of both SG-800 and 40% Pt/C decrease with time, but SG-800 retains 89% of the initial catalytic current after 3.5 h. However, a relative current of only 70% is maintained for commercial Pt/C under the same testing conditions. These data suggest that SG-800 possesses the better duration stability than Pt/C for ORR in an alkaline electrolyte. Combined with high selectivity for ORR and good ability to avoid crossover effects, it is believed that S-doped graphenes prepared by magnesiothermic reduction using Na_2_SO_4_ as the S source is compared to commercial Pt/C for ORR.

## Discussion

In summary, this work has shown an efficient strategy to chemically fix carbon to form valuable, high-quality, doped graphenes, which also can efficiently reduce the emission of CO_2_ into the atmosphere. By magnesiothermic reduction of Na_2_CO_3_ and Na_2_SO_4_ as low-cost, nontoxic carbon and sulfur sources, respectively, S-doped graphenes with few layers have been successfully prepared. The melting Mg can reduce carbon in the oxidation state of +4 in carbonate to form black graphene products. And sulfur in sulfate also can be reduced from its highest oxidation state to lowest valence and hybridized into the carbon sp^2^ framework. The resulting graphenes possess crumpled, sheet-like nanostructures with hierarchically porous textures and the addition of sulfate during the magnesiothermic reaction results in the formation of S-doped graphenes with more distorted nanostructures. The sulfur doping of the graphene framework evidently decreases the ORR onset potentials and increases limited current densities compared with pure graphenes. The resulting S-doped graphenes demonstrate not only high electrocatalytic activity for ORR with a four-electron reaction pathway, but also much better stability and increased tolerance to MeOH crossover effects than commercial Pt/C. The high activity for ORR of S-doped graphenes is mainly ascribed to highly graphitized structures, S-related active sites, and hierarchically porous textures. The present magnesiothermic reduction procedure is being investigated in the preparation of other heteroatom-doped (*e.g.* P and B) graphene materials potentially utilized in the fields of electrocatalysis, batteries, supercapacitors, biosensors, *etc*.

## Methods

### Synthesis of S-doped graphene

Anhydrous Na_2_CO_3_ and Na_2_SO_4_ were used as the C and S sources, respectively, and magnesium (Mg) powder were utilized as the reducing reagent. A calculated amount of these powders were mixed together and then put onto an alumina boat which was inserted into a stainless steel tube. The reaction temperature was controlled to be 700, 800, or 900°C for 1 h at a ramp of 5°C/min under argon (Ar) atmosphere. After cooling to room temperature, the resulting black solids were dispersed in sufficient 10% HCl solution and vigorously stirred for 4 h to remove any inorganic impurities. The carbon was collected by filtration and purified by distilled water and ethanol for three times each. Finally, the filtered product was dried at 80°C overnight and further annealed in Ar atmosphere. And the resulting S-doped graphene was denoted as SG-*X*, where SG is the abbreviation of S-doped graphene and *X* stands for the magnesiothermic reaction temperature in °C. For comparison, pure carbon-based graphene (CG) samples were synthesized by magnesiothermic reduction of Na_2_CO_3_ without any Na_2_SO_4_.

### Structural characterization

Transmission electron microscopy (TEM) images of the graphene were recorded using a JEOL 2010F electron microscope equipped with an energy dispersive spectrometry (EDS) attachment operating at 200 kV. Samples for analysis were deposited on a thin amorphous porous carbon film supported by copper grid from ultrasonically processed ethanol solutions. The morphology of the graphene was observed by scanning electron microscopy (SEM) using a field emission microscope (S-4800, Hitachi). Samples were mounted using a conductive carbon double-sided sticky tape. Raman spectra were recorded on a DXR Raman Microscope (Thermal Scientific Co., USA) with 532 nm excitation length. X-ray powder diffraction (XRD) measurements were carried out on a Rigaku D/MAX-2250 V diffractometer with Cu Kα radiation. X-ray photoelectron spectroscopy (XPS) was recorded with an ESCALAB 250 X-ray photoelectron spectrometer with Al Kα (*hυ* = 1486.6 eV) radiation. The graphene powders were glued onto indium (In) metal particles by pressing for measurements. All spectra were calibrated using 285.0 eV as the line position of adventitious carbon. Nitrogen sorption isotherms were measured using a Micromeritics ASAP 2010 surface area and pore size analyzer at liquid nitrogen temperature (−196°C). Prior to measurement, the hierarchically porous materials were dehydrated under vacuum at 200°C overnight. The specific surface areas were calculated by the Brunauer-Emmett-Teller (BET) method. The total pore volume was calculated from the amount of nitrogen adsorbed at a relative pressure of 0.99. The pore size distribution curves were calculated from the analysis of the desorption branch of the isotherm using the Barrett-Joyner-Halenda (BJH) model.

### Electrode preparation and electrochemical measurements

The graphene sample (5 mg) was dispersed in the mixture of 0.5 mL water and 0.5 mL ethanol under ultrasonic irradiation for *ca*. 2 h. Then 5% Nafion solution (25 μL) was added into the above suspension, and the further ultrasonic treatment was performed until a homogeneous ink was formed. 20 μL ink containing 62.5 μg catalyst was transferred onto the glassy rotating disk electrode with 5 mm diameter, yielding a catalyst level of 0.32 mg/cm^2^. For 40% Pt/C commercial catalyst (JM company), 31.2 μg catalyst was deposited on the glassy rotating disk electrode. The electrode with the catalyst was dried at 50°C which was used as the working electrode for further electrochemical measurements. Electrochemical activity of the working electrode was studied by cyclic voltammetry (CV) and rotating disk electrode (RDE) using a standard three-electrode cell with a Pt plate as the counter electrode and a saturated calomel electrode (SCE) as the reference electrode. An aqueous solution of 0.1 M KOH was used as the electrolyte for the electrochemical studies. The electrochemical cell was controlled by a bipotentiostat (Pine Instrument Co.) equipped with a RDE. Prior to the measurements, the electrolyte was saturated by bubbling O_2_ or N_2_ for 15 min. The working electrode was cycled at least thirty times before the CV data were recorded at a scan rate of 50 mV/s. The RDE measurements were performed at different rotating rates varying from 400 to 2025 rpm with the scan rate of 5 mV/s.

## Author Contributions

J.W. performed the experiments, and wrote the manuscript; R.M., Z.Z. and G.L. analyzed the data; Q.L. designed the experiments; all authors reviewed the manuscript.

## Supplementary Material

Supplementary Information supporting information

## Figures and Tables

**Figure 1:**
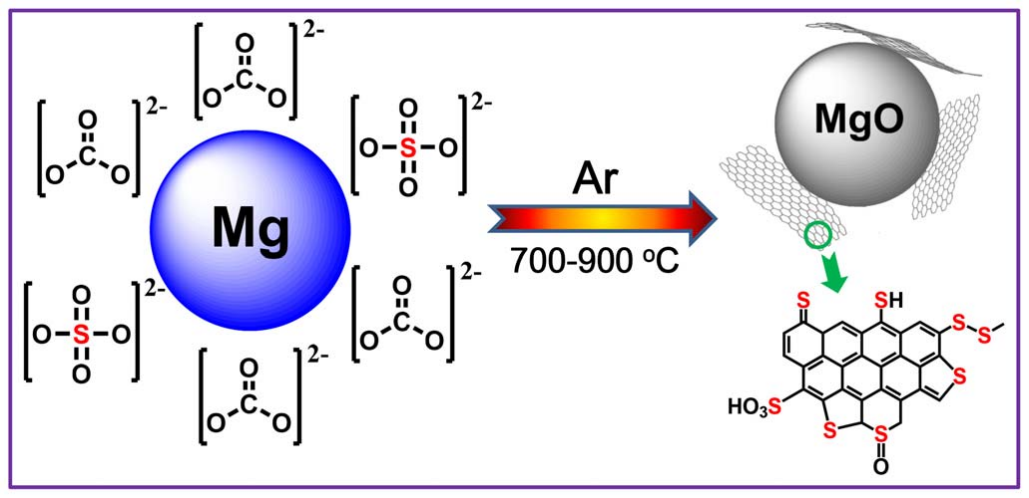
The schematic illustration implying the production of S-doped graphene by magnesiothermic reduction of CO_3_^2−^ in the presence of SO_4_^2−^ in an Ar flow.

**Table 1:** Textural properties of pure graphene and S-doped graphenes prepared at different temperatures

Samples	*S*_BET_ (m^2^/g)	*V*_total_ (cm^3^/g)	*D*_pore_ (nm)
CG-700	233	1.936	3.8
CG-800	239	1.900	3.8
CG-900	222	1.887	3.8
SG-700	188	1.383	3.8
SG-800	199	1.604	3.8
SG-900	190	1.499	3.8
