# Supplementary material for: Magnesiothermic synthesis of sulfur-doped graphene as an efficient metal-free electrocatalyst for oxygen reduction
Source: Sci Rep. 2015 Mar 20;5:9304. doi: 10.1038/srep09304 (PMC4366805; doi:10.1038/srep09304)
Supplement: Supplementary Information — supporting information [file srep09304-s1.pdf]

Supporting Information:

Magnesiothermic synthesis of sulfur-doped graphene as an efficient metal-free electrocatalyst for oxygen reduction

Jiacheng Wang, [1,2,3,]* Ruguang Ma,[1,2,3] Zhenzhen Zhou,[1,2,3] Guanghui Liu,[1,2,3] and Qian Liu[1,2,3,]*

[1]State Key Laboratory of High Performance Ceramics and Superfine Microstructure, Shanghai Institute of Ceramics, Chinese Academy of Sciences, Shanghai 200050, P. R. China.

[2]Innovation Center for Inorganic Materials Genomic Science, Shanghai Institute of Ceramics, Chinese Academy of Sciences, Shanghai 200050, P. R. China.

[3]Shanghai Institute of Materials Genome, Shanghai, P. R. China.

To whom correspondence should be addressed, E-mail: jiacheng.wang@mail.sic.ac.cn; qianliu@sunm.shcnc.ac.cn.

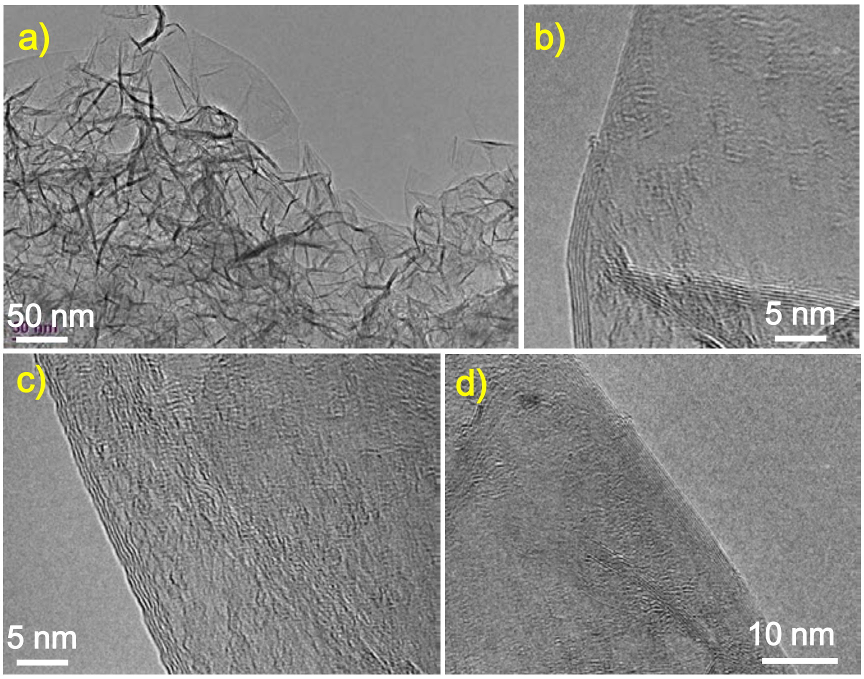

Figure S1. TEM (a) and HR-TEM (b-d) images of CG-800.

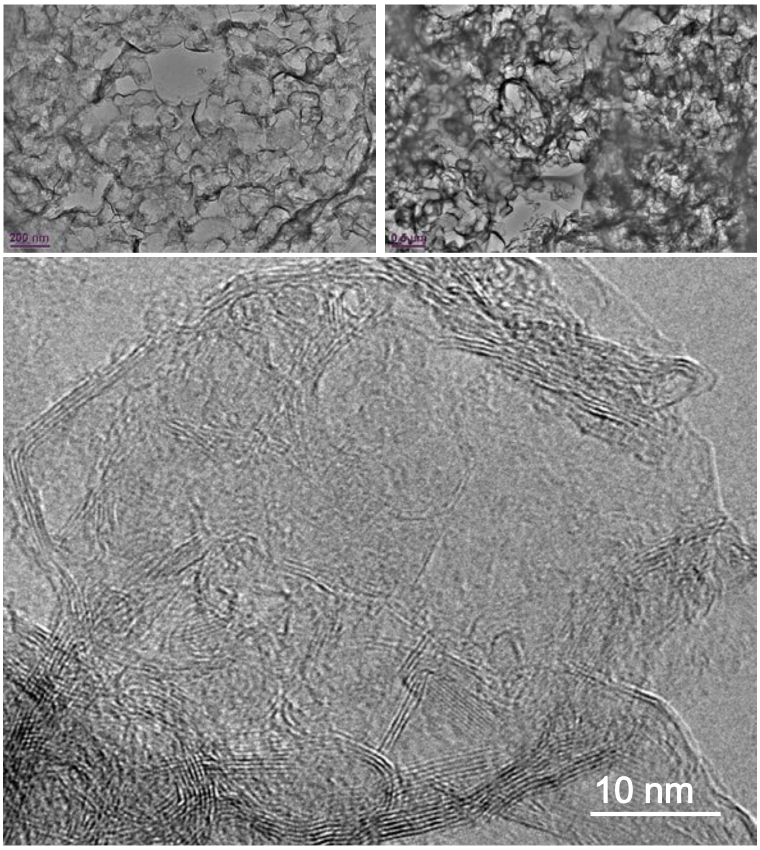

Figure S2. TEM and HRTEM images of SG-800.

Table S1. The C, O, S elemental composition of C-graphene and S-graphenes.

Samples	C (at%)	O (at%)	S (at%)
CG-800	96.6	3.4	0
SG-700	92.6	4.7	2.6
SG-800	94.1	3.6	2.2
SG-900	93.4	4.3	1.8

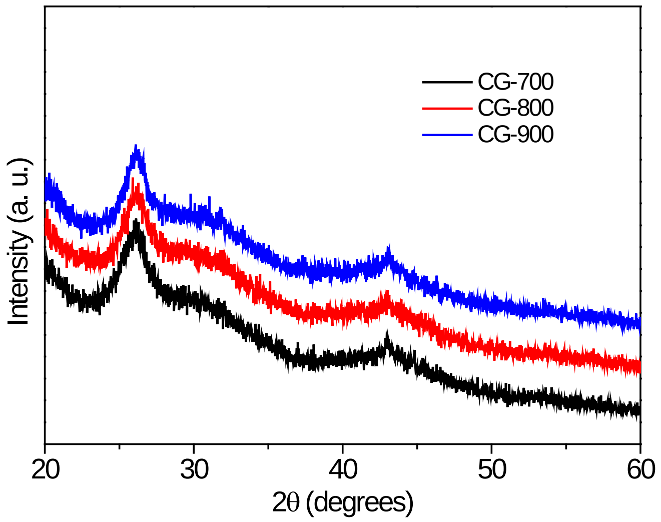

Figure S3. Wide-angle XRD patterns of pure graphene prepared at different temperatures.

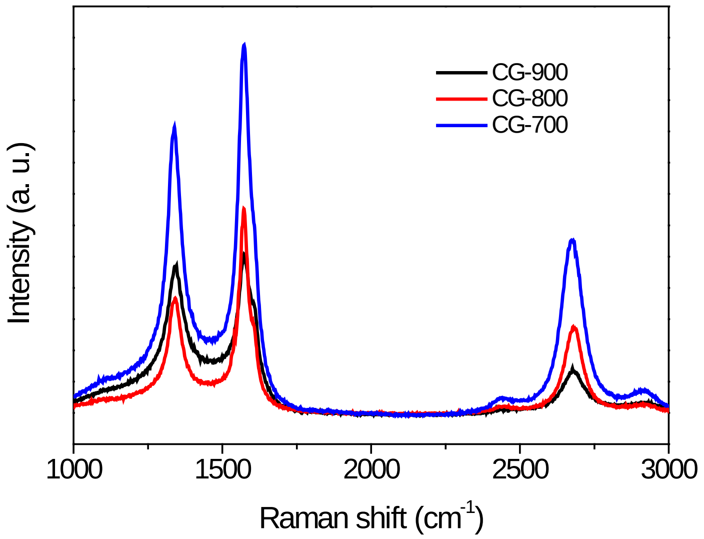

Figure S4. Raman spectra of C-graphene prepared at different temperatures.

Table S2. The data for Raman spectra of CG-800 and S-graphene.

Samples	Position of G	ID/IG	Position of 2D
CG-800	1574	0.51	2681
SG-700	1579.4	0.75	2685
SG-800	1570	0.47	2678
SG-900	1568.4	0.41	2676

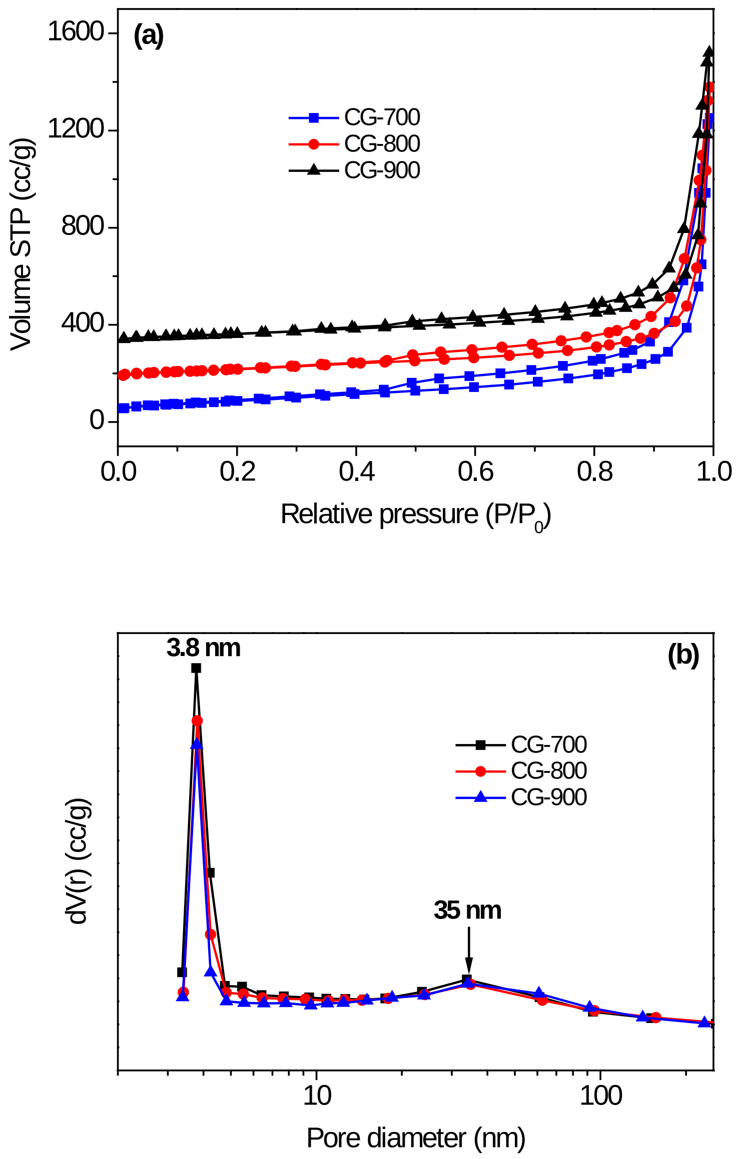

Figure S5. Nitrogen sorption isotherms (a) and pore size distributions (b) of pure C-graphene prepared at different temperatures. The isotherms of CG-800 and CG-900 were shifted up by 150 and 300 units, respectively.

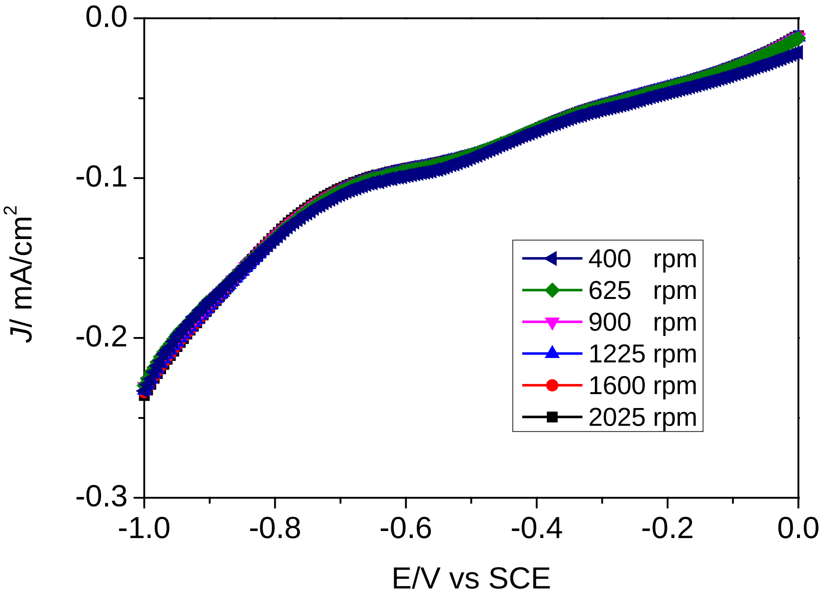

Figure S6. LSV curves of SG-800 in **N_2**-saturated 0.1 M KOH aqueous solution at different rotating speeds of 400~2025 rpm (sweep rate: 5 mV/s).

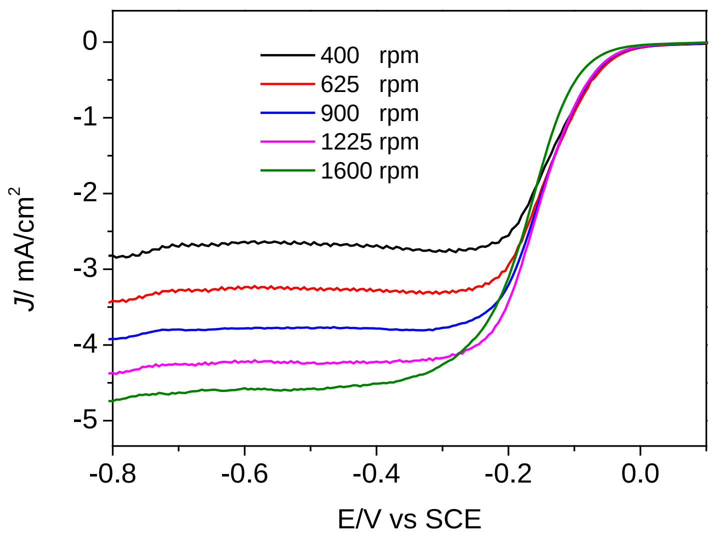

Figure S7. LSVs of 40% Pt/C in O_2-saturated 0.1 M KOH solution at different rotating speeds of 400~1600 rpm (sweep rate: 5 mV/s).

Table S3. Comparison of the ORR properties of S-doped graphene with some other recently reported porous carbon electrocatalysts in O_2-saturated 0.1 M KOH solution at a rotating speed of 1600 rpm.

Samples	Synthesis method	Onset potential (V *vs*. SCE)	Limiting current density (mA/cm^2) at the peak potential	Limiting current density (mA/cm^2) at -1 V (*vs*. SCE)	Ref.
S-doped graphene	Magnesiothermic reduction of Na_2CO_3 and Na_2SO_4	**-0.15**	**-3.3**	**-4.4 at -0.8 V**	**This work**
N-doped porous carbons	Carbonization of ZIF-7/glucose composite	-0.24	-1.5	-4.6	1
N-doped graphene	Annealing of GO under ammonia or N-containing polymer/RGO composite	-0.19	--	-5.6	2
N, S-codoped porous graphene	Annealing of GO, benzyl disulfide, and melamine	-0.11	-3.1	-10 at -0.85 V	3
N, S-codoped porous graphene	Hydrothermal carbonization of glucose and sulfur source, followed by pyrolysis	-0.14	-1.2	-1.82	4
N-doped porous carbons	MOF ZIF-8 as the template and precursor along with furfuryl alcohol and NH_4OH as the secondary carbon and nitrogen source	-0.18	1.36	-4	5
P-doped graphite layers	Pyrolysis of toluene and triphenylphosphine	+0.05	-2.1	-5	6
N-doped ordered mesoporous carbons	Carbonization of nitrogen-containing aromatic compounds using SBA-15 as the template	-0.18	-2.7	-5.9	7
S-doped graphene	Annealing of GO with benzyl disulfide	-0.05	-3.8	-7.2 at -0.85 V	8
Graphene-based carbon nitride sheets	Pyrolysis of ethylenediamine and CCl_4 using mesoporous silica/GO as the template	-0.13	-2.1	-3.7	9
N-doped graphene	Pyrolysis of GO-PANI nanocomposite	-0.16	-1.5	-3.9	10
N-doped graphene	CVD of methane in ammonia	-0.20	-0.60	-0.8 at -0.95 V and 1000 rpm	11
B and N isolate-doped graphitic carbon nanosheets	Pyrolysis of nitrogen-containing anion-exchanged resins containing $[Fe(CN)_6]^{3-}$ and BO_3^-, followed by acid washing	-0.06	-2.4	-3.7 at -0.91 V	12
F-doped carbon black	Heating carbon black in NH4F solution	+0.03	-4.6	-6.1	13
	Pyrolysing P-containing source and carbon source using SBA-15 as a template	-0.16	-2.5	-5.1	14
N-doped carbons	Pyrolysis of gelatin	-0.1	--	-5.4 at -0.7 V	15

N-doped carbon nanocages	Pyrolysis of pyridine using MgO as the template	-0.18	---	----	16
P-doped graphene	Annealing of GO/triphenylphosphine composite	-0.1	-1.7	-4.2	17
N-doped graphene	Annealing of GO-Silica sheets in NH3, followed by removing silica	-0.1	--	---	18
N-doped graphene	Annealing of GO and urea	-0.15	-0.5	-2.75	19
N-doped graphene	Annealing of GO/polydopamine composite	-0.17	-2.5	-4.2	20
N, B-codoped graphene	Annealing of GO in ammonia, and then with H3BO3	-0.15	-2.7	-5.2	21

References

1 Zhang, P. *et al.* ZIF-derived in situ nitrogen-doped porous carbons as efficient metal-free electrocatalysts for oxygen reduction reaction. *Energy Environ. Sci.* **7**, 442-450 (2014).

2 KokáPoh, C. Exploration of the active center structure of nitrogen-doped graphene-based catalysts for oxygen reduction reaction. *Energy Environ. Sci.* **5**, 7936-7942 (2012).

3 Liang, J., Jiao, Y., Jaroniec, M. & Qiao, S. Z. Sulfur and Nitrogen Dual‐Doped Mesoporous Graphene Electrocatalyst for Oxygen Reduction with Synergistically Enhanced Performance. *Angew. Chem. Int. Ed.* **51**, 11496-11500 (2012).

4 Wohlgemuth, S.-A. *et al.* A one-pot hydrothermal synthesis of sulfur and nitrogen doped carbon aerogels with enhanced electrocatalytic activity in the oxygen reduction reaction. *Green Chem.* **14**, 1515-1523 (2012).

5 Aijaz, A., Fujiwara, N. & Xu, Q. From Metal-Organic Framework to Nitrogen-Decorated Nanoporous Carbons: Superior CO2 Uptake and Highly Efficient Catalytic Oxygen Reduction. *J. Am. Chem. Soc.* **136**, 6790-6793 (2014).

6 Liu, Z. W. *et al.* Phosphorus‐Doped Graphite Layers with High Electrocatalytic Activity for the O2 Reduction in an Alkaline Medium. *Angew. Chem.* **123**, 3315-3319 (2011).

7 Liu, R., Wu, D., Feng, X. & Müllen, K. Nitrogen‐Doped Ordered Mesoporous Graphitic Arrays with High Electrocatalytic Activity for Oxygen Reduction. *Angewandte Chemie* **122**, 2619-2623 (2010).

8 Yang, Z. *et al.* Sulfur-doped graphene as an efficient metal-free cathode catalyst for oxygen reduction. *ACS Nano* **6**, 205-211 (2011).

9 Yang, S., Feng, X., Wang, X. & Müllen, K. Graphene‐Based Carbon Nitride Nanosheets as Efficient Metal‐Free Electrocatalysts for Oxygen Reduction Reactions. *Angew. Chem. Int. Ed.* **50**, 5339-5343 (2011).

10 Lin, Z. *et al.* Simple preparation of nanoporous few-layer nitrogen-doped graphene for use as an efficient electrocatalyst for oxygen reduction and oxygen evolution reactions. *Carbon* **53**, 130-136 (2013).

11 Qu, L., Liu, Y., Baek, J.-B. & Dai, L. Nitrogen-doped graphene as efficient metal-free electrocatalyst for oxygen reduction in fuel cells. *ACS Nano* **4**, 1321-1326 (2010).

12 Wang, L. *et al.* B and N isolate-doped graphitic carbon nanosheets from nitrogen-containing ion-exchanged resins for enhanced oxygen reduction. *Sci. Rep.* **4**, 5184 (2014).

13 Sun, X. *et al.* Fluorine-Doped Carbon Blacks: Highly Efficient Metal-Free Electrocatalysts for Oxygen Reduction Reaction. *ACS Catal.* **3**, 1726-1729 (2013).

14 Yang, D.-S. *et al.* Phosphorus-doped ordered mesoporous carbons with different lengths as efficient metal-free electrocatalysts for oxygen reduction reaction in alkaline media. *J. Am. Chem. Soc.* **134**, 16127-16130 (2012).

15 Nam, G. *et al.* Metal-Free Ketjenblack Incorporated Nitrogen-Doped Carbon Sheets Derived from Gelatin as Oxygen Reduction Catalysts. *Nano Lett.* **14**, 1870-1876 (2014).

16 Chen, S. *et al.* Nitrogen‐Doped Carbon Nanocages as Efficient Metal‐Free Electrocatalysts for Oxygen Reduction Reaction. *Adv. Mater.* **24**, 5593-5597 (2012).

17 Zhang, C. *et al.* Synthesis of Phosphorus‐Doped Graphene and its Multifunctional Applications for Oxygen Reduction Reaction and Lithium Ion Batteries. *Advanced Materials* **25**, 4932-4937 (2013).

18 Yang, S. *et al.* Efficient Synthesis of Heteroatom (N or S) - Doped Graphene Based on Ultrathin Graphene Oxide - Porous Silica Sheets for Oxygen Reduction Reactions. *Adv. Funct. Mater.* **22**, 3634-3640 (2012).

19 Lin, Z. *et al.* Facile Synthesis of Nitrogen - Doped Graphene via Pyrolysis of Graphene Oxide and Urea, and its Electrocatalytic Activity toward the Oxygen - Reduction Reaction. *Advanced Energy Materials* **2**, 884-888 (2012).

20 Cong, H.-P., Wang, P., Gong, M. & Yu, S.-H. Facile synthesis of mesoporous nitrogen-doped graphene: An efficient methanol–tolerant cathodic catalyst for oxygen reduction reaction. *Nano Energy* **3**, 55-63 (2014).

21 Zheng, Y. *et al.* Two - Step Boron and Nitrogen Doping in Graphene for Enhanced Synergistic Catalysis. *Angew. Chem.* **125**, 3192-3198 (2013).
